# Changes to the gut microbiota of a wild juvenile passerine in a multidimensional urban mosaic

**DOI:** 10.1038/s41598-022-10734-7

**Published:** 2022-04-27

**Authors:** Öncü Maraci, Michela Corsini, Anna Antonatou-Papaioannou, Sebastian Jünemann, Joanna Sudyka, Irene Di Lecce, Barbara A. Caspers, Marta Szulkin

**Affiliations:** 1 Department of Behavioural Ecology, Bielefeld University, Konsequenz 45, 33619 Bielefeld, Germany; 2 Centre of New Technologies, University of Warsaw, Banacha Street 2C, 02-097 Warsaw, Poland; 3 Evolutionary Biology, Bielefeld University, Universitätsstrasse 25, 33615 Bielefeld, Germany; 4 Institute of Biology-Zoology, Freie Universität Berlin, Köning-Luise-Str. 1-3, 14195 Berlin, Germany; 5 Faculty of Technology, Bielefeld University, Universitätsstrasse 25, 33615 Bielefeld, Germany; 6 Center for Biotechnology (CeBiTec), Bielefeld University, Sequenz 1, 33615 Bielefeld, Germany; 7 Institute of Environmental Sciences, Jagiellonian University, ul. Gronostajowa 7, 30-387 Kraków, Poland

**Keywords:** Ecology, Evolution, Microbiology

## Abstract

Urbanisation is a major anthropogenic perturbation presenting novel ecological and evolutionary challenges to wild populations. Symbiotic microorganisms residing in the gastrointestinal tracts (gut) of vertebrates have mutual connections with host physiology and respond quickly to environmental alterations. However, the impact of anthropogenic changes and urbanisation on the gut microbiota remains poorly understood, especially in early development. To address this knowledge gap, we characterised the gut microbiota of juvenile great tits (*Parus major*) reared in artificial nestboxes and in natural cavities in an urban mosaic, employing two distinct frameworks characterising the urban space. Microbial diversity was influenced by cavity type. Alpha diversity was affected by the amount of impervious surface surrounding the breeding location, and positively correlated with tree cover density. Community composition differed between urban and rural sites: these alterations covaried with sound pollution and distance to the city centre. Overall, the microbial communities reflect and are possibly influenced by the heterogeneous environmental modifications that are typical of the urban space. Strikingly, the choice of framework and environmental variables characterising the urban space can influence the outcomes of such ecological studies. Our results open new perspectives to investigate the impact of microbial symbionts on the adaptive capacity of their hosts.

## Introduction

In today’s fast-changing world, cities are expanding at an unprecedented rate. Urban growth modifies a wide range of biotic and abiotic ecosystem properties, thereby presenting new ecological and evolutionary challenges for wild populations^1^. Given the scales and magnitudes of these environmental changes, adapting to human-altered environments is a challenging task for animals, and populations living in the urban space are often found to differ from their rural counterparts in terms of their morphological, physiological, and behavioural traits^2–4^.

The radically transformed environment present in the urban space is expected to also influence the symbiotic interactions between microorganisms and animals. The gut microbiome is increasingly recognized as a key player in several aspects of host physiology^5,6^. Indeed, as urbanisation alters the distribution of multiple environmental variables^1^ as well as various traits of individual hosts, these changes altogether shape the assembly, taxonomic diversity, and functioning of animal-associated microorganisms via different mechanisms^7^. For example, urbanisation can change the environmental pool of microorganisms, host diet^8^, and host physiology^9^. All these changes can potentially translate into microbial community shifts in the gut.

An adequate definition of the urban space is essential when addressing the potential impact of urban change on animal-microbe symbiosis. Classical frameworks describing urban areas often rely on a threshold-based human density value that overlaps with a city administrative borders^1^. Accordingly, cities consist of a core area in which the majority of the population lives (urban site), and peri-urban (rural) sites that accommodate relatively smaller proportions of human inhabitants. This dichotomous categorization can capture some alterations that are directly related to human activities and, consequently change gradually from the core of a city to its periphery, for example in terms of air pollution, sound pollution and light pollution^10^. However, the spatial units defined by humans rarely consist of homogeneous habitats. Rather, cities are characterised by mosaics of urban landscapes; these mosaics vary in terms of actual surface use and accompanying fine-scale differences in habitat type and environmental characteristics that are not entirely reflected in a simple urban/rural categorization^1^. In this light, defining the urban space by quantifying the actual changes in its physical attributes, such as the percentage of impervious surface area (ISA) that characterises the urban space, might be a biologically more relevant approach to characterise variation in the gut microbiota. Consequently, the methodological approach used to characterize the urban space (e.g. dichotomous urban–rural comparisons vs. those reflective of urban heterogeneity) has the potential to influence the outcome of studies investigating the impacts of urbanisation on ecological interactions.

Our knowledge of how avian gut microbiota changes in urban habitats comes from a handful of studies with mixed results: thus, gut microbial diversity was found to be lower in urban populations of house sparrow (*Passer domesticus*)^11^, American white ibises (*Eudocimus albus*)^12^ and herring gulls (*Larus argentatus*)^13^, while it was higher in city-dwelling white-crowned sparrows (*Zonotrichia leucophrys*)^14,15^. Another knowledge gap related to our understanding of animal-microbe symbiosis in the urban space is associated with the fact that all studies published to date were performed on adult birds and reptiles; thus, there are currently no data exploring the extent to which the gut microbiome of birds hatching and growing in urban environments are dissimilar to birds developing in natural forests. This information gap is particularly striking as the microbial colonies established in early life are known to have vital functions in the developmental trajectory of an individual, affecting the survival and fitness of vertebrates^16,17^. Also pertinent to the theme of juvenile development and life-long physiological homeostasis is the fact that cavity-breeding songbirds in urban environments are known to occupy both nestboxes and natural cavities^18^. But it should be noted that nestboxes, whose supplementation in urban environments is essential to provide breeding cavities for cavity-nesting birds, provide different microclimatic conditions than natural cavities^18,19^, possibly impacting the shaping of the avian microbiome. However, the impact of the use of artificial breeding cavities such as nestboxes on the gut microbiota has never been investigated. Understanding these overlooked aspects of songbird biology can advance our understanding of urban-driven responses in animal-associated microbial communities, and broaden our perspective on the evolutionary consequences of animal-microbe symbiosis.

To address these knowledge gaps, we collected faecal samples from great tit (*Parus major*) nestlings non-invasively and investigated changes in the taxonomic diversity and community composition of faecal gut microbiota sampled across a heterogeneous urban landscape in Warsaw, Poland. These samples represent an urban mosaic ranging from natural rural habitats to highly modified urban areas, consequently exhibiting profound variation in multiple environmental parameters and in the rearing environment (natural cavities and nestboxes). Such sampling strategy allowed us to test whether (1) microbial community diversity and composition are influenced by breeding cavity type (artificial nestbox or natural cavity), (2) microbiota differs in terms of diversity and community composition as described by two different frameworks defining the urban environment (urban/rural dichotomy *vs.* gradual change in ISA) and finally, whether (3) changes in microbial alpha and beta diversity covary with distinct environmental variables characterising the urban space.

## Results

**Figure 1:**
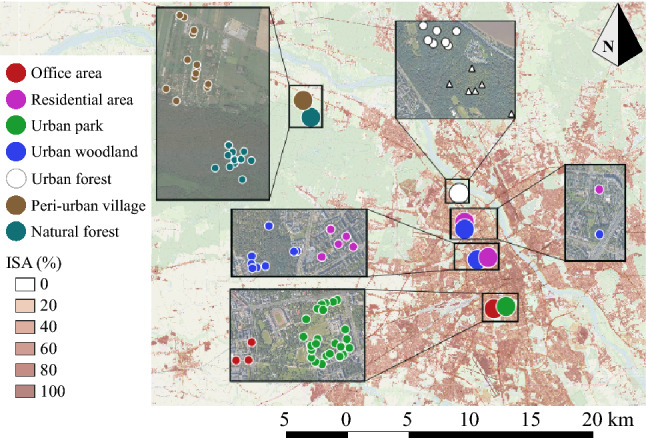
Study site locations in the urban mosaic of the capital city of Warsaw, Poland. The locations include an office area, two residential areas, an urban park, two urban woodlands, an urban forest, a peri-urban village and a natural forest. The impervious surface area (ISA, in %), shown here as the original map layer, is further described and used for analysis in Section 2.2.3. A zoom on each study site visualizes the locations of nestboxes (dots) and natural cavities (triangles). The figure was generated using the QGIS software (v. 3.10)^86^.

We collected faecal samples from 15-day old great tit nestlings hatched in nine different sites, located within and outside of the capital city of Warsaw in Poland (Fig. 1). We characterised gut microbial communities using 16s ribosomal RNA (rRNA) gene sequencing. After quality-filtering, the resulting dataset consisted of 82 biological samples collected from 80 nests and 1090 OTUs with a total read count of 1,277,151 (Minimum = 937, Maximum = 132,856, Average = 15,575.01, SD = 19,259.16). Based on the OTU accumulation curve, our sample size is sufficient for an accurate estimation of microbial communities (Supplementary Figure 1). These OTUs were represented by 19 different microbial phyla, three of which constituted 96.9% of the OTUs based on their total relative abundances: Firmicutes (69.5%), Proteobacteria (18.6%) and Actinobacteria (8.8%).

### Microbial community diversity and composition in relation to cavity type

**Figure 2:**
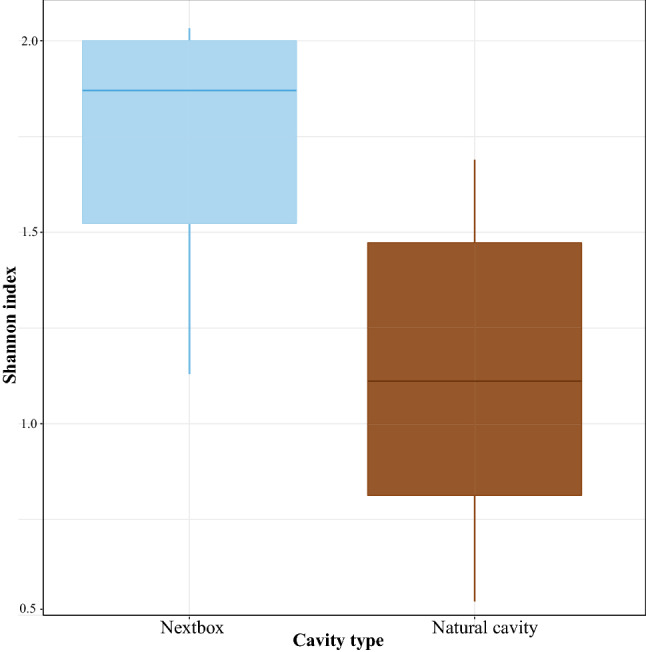
Microbiota alpha-diversity based on Shannon’s diversity collected from nestlings reared in nestboxes and natural cavities. The figure was generated in R version 4.0.0^68^.

To understand the impact of cavity type on microbial communities, we compared the samples collected from nestboxes (N = 7) and those collected from natural cavities (N = 6) located in the same urban forest. While Shannon’s diversity was lower in nestlings reared in natural cavities (linear model; β = − 0.60 ± 0.22, 95% CI [− 1.09; − 0.11], *p* = 0.021; Fig. 2), two other alpha diversity metrics (number of observed OTUs ad Faith’s phylogenetic diversity index) did not change between the two cavity types (Supplementary Table 1). Similarly, microbial composition did not differ between nestlings originating from nestboxes and natural cavities (PERMANOVA: Bray–Curtis: pseudo-F = 0.25, *p* = 1; weighted UniFrac: pseudo-F = 0.53, *p* = 0.92).

### The impact of urbanization on microbial community diversity and composition

**Figure 3:**
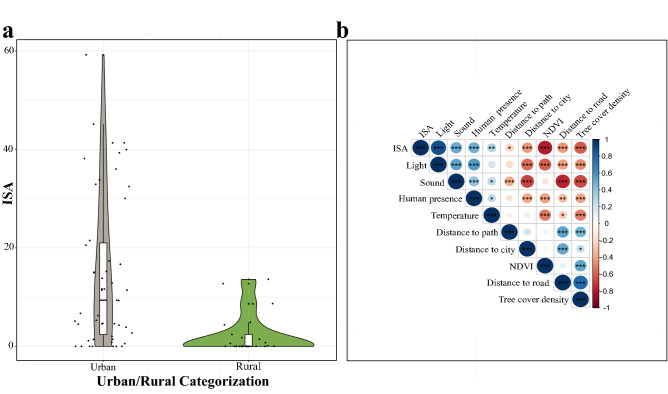
(**a**) Violin plots with embedded box plots comparing the distribution of ISA percentages in urban and rural sites. The line within the box plots indicates the median and the lower and upper boundary of the boxes indicates the 25th and 75th percentile, respectively. Whiskers above and below the boxes correspond to 1.5 times the inter-quartile range (IQR) above and below the 25th and 75th percentile, respectively. (**b**) Correlation between ISA and environmental variables. The significance was determined based on Pearson’s correlation tests, at *p*-values ≤ 0.05 (*), *p* ≤ 0.01 (**), and *p* ≤ 0.001 (***). The sizes of the circles are proportional to Pearson’s correlation coefficients. The figure was generated in R version 4.0.0^68^.

**Figure 4:**
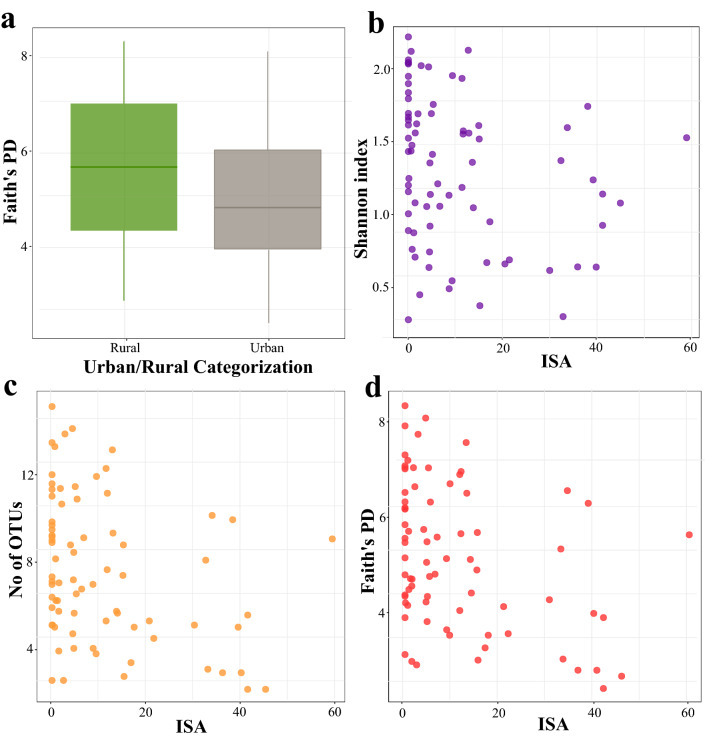
Alpha diversity across the urban mosaic. (**a**) Boxplot of Faith's phylogenetic diversity of nestlings originating from urban and rural sites. Scatter plots report the relationship between ISA and (**b**) Shannon’s diversity index (**c**) The number of observed OTUs, and (**d**) Faith's phylogenetic diversity. The figure was generated in R version 4.0.0^68^.

To understand the impact of urbanization on the gut microbiota, we defined the urban space using two different frameworks. Under the *first framework of urbanisation*, each nest was categorised depending on whether it was located within the administrative limits of the city (urban) or outside of these limits (rural, Fig. 1). This aligns our findings with frequent partitioning of phenotypic^20^ and genetic variation^21^. However, the actual surface use patterns measured by the percentage of Impervious Surface Area around each nest location (ISA) show substantial heterogeneity within urban and rural sites (Fig. 3a, Supplementary Table 2). Furthermore, ISA correlated with all the environmental parameters used in the study (Fig. 3b, Supplementary Table 3). Taken together, ISA can be used as an urban metric to quantify actual surface use intensity, and to capture accompanying fine-scale differences in several environmental parameters that often covary^1^ or interact with each other across the urban mosaic. Consequently, under the *second framework of urbanisation*, we used the percentage of ISA surrounding the respective location of every nest analysed in the study. We investigated the impact of urbanisation on alpha diversity as measured by Shannon’s diversity, the number of observed OTUs, and Faith’s phylogenetic diversity index with linear models (LMs). When analysed based on administrative boundaries (urban vs. rural), Faith’s phylogenetic diversity index was lower in urban sites (β = − 0.72 ± 0.36, 95% CI [− 1.44 to − 0.01], *p* = 0.048, Fig. 4a), while the other alpha diversity metrics did not differ between hosts from urban and rural sites (Supplementary Table 1). When we analysed the covariation of diversity metrics with ISA (as a continuous variable), Faith’s phylogenetic diversity index was also lower in urban sites (β = − 0.04 ± 0.01, 95% CI [− 0.06 to − 0.02], *p* = 0.001). Importantly however, the remaining alpha diversity metrics covaried significantly and negatively with ISA (Shannon’s diversity: β = − 0.01 ± 0.00, 95% CI [− 0.02 to − 0.00], *p* = 0.019; the number of observed OTUs: β = − 0.08 ± 0.03, 95% CI [− 0.13 to − 0.03], *p* = 0.004; Fig. 4b–d, Supplementary Table 1). Importantly, all fitted linear models were tested for spatial autocorrelation by Moran’s I test and showed no evidence for geographical structuring of alpha diversity (see Supplementary Table 1 for *p* values). Moreover, as there was temporal variation in nest initiation, sampling dates differed between the nests, spanning from the 19th of May 2018 to the 20th of July 2018. We examined whether the temporal variation in sampling affected alpha diversity using LMs. However, the number of days between initiation of the study and sample collection did not affect Shannon’s diversity index (*p* = 0.57), the number of observed OTUs (*p* = 0.09), and Faith’s phylogenetic diversity index (*p* = 0.86).

**Figure 5:**
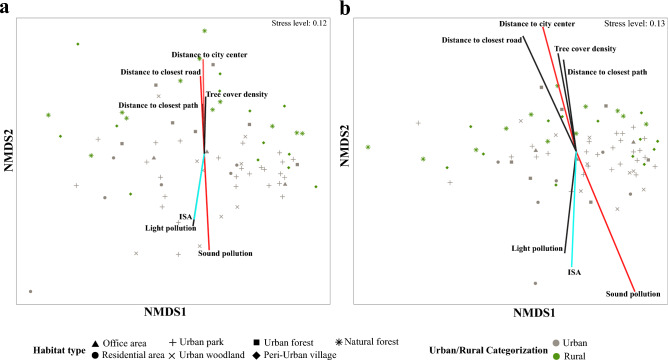
nMDS plots of microbial dissimilarities based on (**a**) Bray–Curtis and (**b**) Weighted UniFrac distances among samples collected from different sampling sites located in urban and rural areas. The lengths of vectors are proportional to their predictive strength, determined based on *Envfit*. All variables that were significant based on at least one test are represented (The significance was determined based on being included in the best* BIOENV* model or at p-values ≤ 0.05 in *Envfit* or partial Mantel test). The parameters considered significant in all tests are coloured in red. The figure was generated in R version 4.0.0^68^.

The nMDS plots based on Bray–Curtis (Fig. 5a) and Weighted UniFrac (Fig. 5b) dissimilarities of the gut microbiota revealed urban-driven shifts in microbial composition. We statistically tested whether microbial communities differ between nestlings originating from urban and rural areas, different study sites, or with changing ISA levels (fitted as a continuous variable) by PERMANOVA models based on Bray–Curtis and the weighted UniFrac dissimilarities. According to the model based on Bray–Curtis dissimilarity, microbial composition differed between urban and rural areas (pseudo-F = 1.66, *p* = 0.044) and sampling sites (pseudo-F = 1.28, *p* = 0.019), but not with changing ISA percentages (pseudo-F = 1.08, *p* = 0.284). PERMANOVA based on Weighted UniFrac distances detected differences in microbial composition between urban and rural sites (pseudo-F = 2.58, *p* = 0.030), but not between sampling sites (pseudo-F = 1.17, *p* = 0.202) or with changing ISA percentages (pseudo-F = 1.27, *p* = 0.218). We also tested the homogeneity of group dispersion between urban and rural sites. PERMDISP based on Bray–Curtis dissimilarity showed homogeneous dispersions for urban and rural samples, indicating that the significant PERMANOVA results were not caused by differences in dispersion among the groups. However, PERMDISP based on weighted UniFrac distances revealed that microbial variation in rural sites is significantly higher than in urban sites (the average distance from the centroids was 0.144 ± 0.006 and 0.125 ± 0.004 and for rural and urban areas, respectively, *p* = 0.035). Therefore, the significant between-group differences detected by PERMANOVA might reflect differences in location, dispersion, or a combination of the two. There was no significant impact of sampling time in our PERMANOVA model (based on Bray–Curtis dissimilarity: *p* = 0.30; based on Weighted UniFrac: *p* = 0.12).

**Figure 6:**
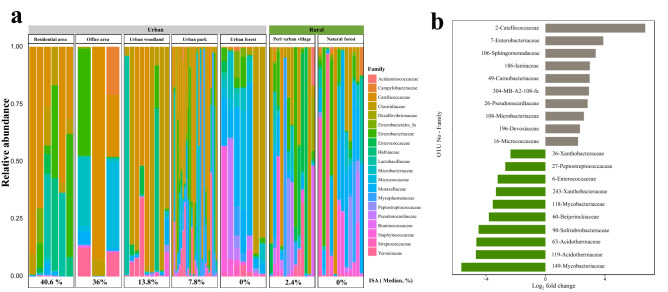
(**a**) The relative abundances of microbial families in gut samples from different sampling sites located in urban and rural territories. Only the 20 families with the highest relative abundances are reported. (**b**) Differentially abundant OTUs between urban and rural samples. OTUs with a log_2_-fold change larger than zero are more abundant in urban territories (grey bars), while OTUs with a log2-fold change smaller than zero are more abundant in rural territories (green bars). The family level taxonomy of each OTU is indicated. The figure was generated in R version 4.0.0^68^.

The stacked bar plot revealed prominent taxonomic and compositional differences between urban and rural hosts (Fig. 6a). To determine differentially abundant OTUs between the urban and rural territories, we employed DESeq2 analysis^22^. Overall, we found 20 differentially abundant OTUs (Fig. 6b, Supplementary Table 4). Of these, ten were significantly more abundant in the rural territories, and ten were significantly more abundant in urban territories. Urban hosts exhibited higher abundances of an OTU belonging to the potentially pathogenic microbial family, *Enterobacteriaceae*^23,24^.

### Interactive effects of spatial and environmental parameters on microbial change

While nesting sites are here referred to as located in urban or rural sites or areas with varying degrees of ISA, each nestbox is also embedded in a complex web of specific environmental and spatial parameters characterising the urban mosaic; these parameters are defined herein as the distance to city centre, closest road and closest path, light pollution, sound pollution, human presence, temperature, tree cover density, and NDVI. These environmental parameters differed between the urban and rural sites (except for the distance to the closest path, NDVI and tree cover density; Supplementary Table 5), and were correlated with ISA (Supplementary Table 3).

**Figure 7:**
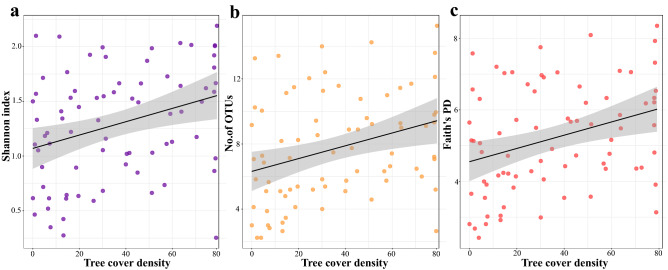
Scatter plots reporting the relationship between tree cover density and (**a**) Shannon’s diversity index and (**b**) the number of observed OTUs, (**c**) Faith’s phylogenetic diversity. The figure was generated in R version 4.0.0^68^.

We investigated which environmental and spatial variables best predicted the microbial alpha diversity (diversity within samples) as measured by Shannon’s diversity, the number of OTUs, and Faith’s phylogenetic diversity index. As multiple environmental and spatial variables were correlated with each other (Fig. 3b, Supplementary Table 3), we first examined multicollinearity among all variables and sequentially excluded predictors based on VIF values and biological relevance when analysing predictors of alpha diversity. Our final models included five predictors: distance to the city centre, human presence, temperature, tree cover density and distance to the closest path (Supplementary Table 6). Among these predictors, only tree cover density was positively associated with Shannon’s diversity (LMM, β = 0.18 ± 0.09, 95% CI [0.00–0.36], *p* = 0.047), the number of observed OTUs (β = 1.2 ± 0.59, 95% CI [0.03 to 2.37], *p* = 0.045) and Faith’s phylogenetic diversity (β = 0.56 ± 0.28, 95% CI [0.01 to 1.11], *p* = 0.048) (Fig. 7).

Before identifying the strongest predictors of between-group microbial dissimilarities, we assessed the spatial autocorrelation of beta diversity by Mantel test. The distance matrix of the geographical coordinates of the sampling sites was correlated with Bray–Curtis dissimilarity matrix (R = 0.054, *p* = 0.041), but not with the weighted UniFrac dissimilarity matrix (R = 0.018, *p* = 0.250). We further tested the associations between all environmental variables and beta diversity by employing different analytical methods to obtain a coherent vision of the urban-driven variation in gut microbiota. We applied a partial mantel test to investigate the correlations between microbial dissimilarity matrices and the distance matrices of the environmental variables, controlling for the effect of the distance matrix of the geographical coordinates of the sampling sites. We found that sound pollution, distance to the city center, distance to the closest road and tree cover density were significantly correlated with Bray–Curtis and Weighted UniFrac dissimilarities (Table 1). All other parameters were found to be nonsignificant in the partial Mantel tests (Table 1). Based on *BIOENV*, the best model with the strongest relationship with Bray–Curtis dissimilarity matrix (R = 0.209) similarly contained sound pollution, the distance to the city centre, the distance to the closest road and light pollution (Table 1). However, the best *BIOENV* model based on weighted UniFrac distance (R = 0.200) contained only sound pollution and distance to the city centre. According to *Envfit*, patterns observed in the Bray–Curtis and Weighted UniFrac ordination plot were significantly associated with sound pollution, the distance to the city centre, and distance to the closest road, light pollution, tree cover density, and the distance to the closest path,) but not with temperature, NDVI or human presence (Table 1). Overall, there was coherent and conclusive support across all models for sound pollution and distance to the city centre acting as the most important drivers of juvenile gut microbiota variation across the urban mosaic (Fig. 5).

**Table 1:** Summary of the statistical tests used to investigate the interaction between environmental variables and beta diversity. The bold significance was determined based on *Envfit* or partial mantel test, at p-values ≤ 0.05 or being selected by the best *BIOENV* model.

Environmental parameter	Envfit				Partial mantel				BIOENV	
	BC		WU		BC		WU		In the best model	
	r2	*p*	r2	*p*	r	*p*	r	*p*	BC	WU
**Sound pollution**	**0.33**	**0.0001**	**0.25**	**0.0003**	**0.18**	**0.0003**	**0.20**	**0.0005**	**Yes**	**Yes**
**Distance to the city center**	**0.32**	**0.0001**	**0.18**	**0.0005**	**0.15**	**0.0032**	**0.14**	**0.0046**	**Yes**	**Yes**
**Distance to the closest road**	**0.22**	**0.0005**	**0.18**	**0.0012**	**0.11**	**0.0353**	**0.15**	**0.0083**	**Yes**	No
Tree cover density	**0.12**	**0.0108**	**0.11**	**0.0125**	**0.08**	**0.0139**	**0.07**	**0.0290**	No	No
Light pollution	**0.20**	**0.0004**	**0.12**	**0.0087**	0.02	0.3226	− 0.01	0.5561	**Yes**	No
Distance to the closest path	**0.09**	**0.0318**	**0.10**	**0.0257**	0.06	0.1412	0.05	0.1787	No	No
Temperature	0.05	0.1402	0.01	0.6039	− 0.19	0.6367	0.04	0.2148	No	No
NDVI	0.03	0.2910	0.03	0.3023	0.00	0.4968	− 0.01	0.5499	No	No
Human presence	0.03	0.3373	0.03	0.3769	0.00	0.4415	− 0.01	0.5540	No	No

## Discussion

### Cavity type influences microbial diversity but not community composition

Our dataset, albeit small, indicated that Shannon’s diversity, one of the alpha diversity metrics investigated in the study, was higher in nestlings reared in artificial nestboxes, relative to those reared in natural cavities. Since the nestboxes and natural cavities located on a homogenous urban forest (see^18^), the observed changes cannot be justified by differences in food sources or nest material. Human-made nestboxes are frequently used in ecological studies, as they are convenient substitutes for natural cavities. However, they differ from natural cavities in various aspects, exhibiting very different humidity ranges relative to natural cavities and providing poorer insulation, leading to prominent fluctuations in daily ambient temperatures across 24 h^19^. While the gastrointestinal ecosystem is relatively well-regulated and is not expected to exhibit prominent alterations in response to moderate fluctuations in ambient conditions in endothermic animals, young nestlings of altricial passerines cannot regulate their body temperatures^25^. Therefore, even moderate fluctuations in microclimatic conditions can trigger physiologic stress in nestlings and consequently, influence the immune system^26^. Although we still know very little about how these alterations translate into gut microbiota^27^, some studies revealed an increased microbial diversity with elevated temperatures^28^, probably due to suppression of immune response. Another explanation is that such fluctuations can influence nestling development and differential growth rates between nestlings from different cavity types can ultimately affect the microbial assembly. At the same time, there were no difference in the species richness, phylogenetic diversity, or composition of microbial communities of the nestlings reared in nestboxes and natural cavities. It is important to note that the novel finding regarding increased microbial diversity in nestboxes relies on a small dataset and should be confirmed by larger datasets.

### Alterations in the diversity and taxonomic composition of gut microbiota in the urban space

Gut microbial communities differed across the urban mosaic, and we demonstrate that the type of framework used to define the urban space impacts analytical outcomes and results interpretation. Remarkably, occupying urban or rural habitats per se (strictly based on urban/rural administrative definitions) does little to explain changes in alpha diversity. Instead, microbial diversity was found to be lower in highly urbanised space, and was correlated with actual land-use intensity, as measured by ISA (Fig. 4). Thus, the observed alterations in alpha diversity are probably more strongly associated with fine-scale variation in the local habitat, which shows remarkable heterogeneity within the urban mosaic, rather than with factors broadly related to human activities or the geographical locations of the nests. Thus, urban spatial heterogeneity can influence the gut microbiota diversity via different mechanisms (see below). Earlier studies investigating how microbial alpha diversity varies in the urban space have revealed contradictory results. For example, the gut microbial diversity was higher in urban populations of white-crowned sparrows than in their rural counterparts, yet the change was not strongly associated with impervious cover^14,15^, which is inconsistent with our findings. In contrast, adult house sparrows had reduced alpha diversity in urbanised habitats with more than 10% built-up areas^11,29^. Similarly, herring gulls exhibited reduced diversity in highly urbanised areas as determined by the human population^13^. Furthermore, alpha diversity did not differ between urban and rural populations in juvenile house sparrows^29^ or was not associated with the percentage of urban land cover in American white ibises^12^. Based on the results reported in this study, we posit that these discrepancies may be driven by differences in the criteria used to define the urban space. Alternatively, but not exclusively, biological differences originating from the taxonomy and life-history stage of the host may also play a role in these discrepancies. For example, avian species with higher tolerance to anthropogenic food might exhibit higher alpha diversity in urban areas.

We demonstrate that microbial community composition changes with contrasting land-use patterns. Importantly, and in contrast to the results obtained for alpha diversity, these alterations were prominent only under the urban/rural distinction reflected by administrative borders, indicating that differences in community composition were largely determined by whether the nestling lived in a densely populated urban site. Strikingly, the urban hosts were enriched by a potentially pathogenic microbial family, *Enterobacteriaceae*^23,30^. A higher prevalence of *Enterobacteriaceae* has been associated with dysbiosis in mice^31^ and higher mortality rates in ostriches^32^. Similarly, urban populations of American white ibises were found to have lower microbial diversity and were more susceptible to Salmonella infections than their rural counterparts^12^. Collectively, these findings indicate that pathogen susceptibility increases in densely populated urban sites, potentially adversely influencing the overall health and fitness of animal hosts. However, it is important to note that the differential abundance analysis did not account for the nonindependence of the samples collected from the same site. Therefore, our results might be related to the increased prevalence of this pathogenic taxon in particular sites rather than overall urban populations.

Taken together, our findings revealed that diversity and composition of the microbial communities of juvenile great tits change across an urban mosaic. Furthermore, using different frameworks to describe the urban space leads to the capturing of different aspects of variation in microbial communities. The initial microbial colonies inhabited by animal hosts in early life are not only critical for the establishment of healthy gut microbiota but also have crucial functions, such as the programming of the immune system^33^, and are involved in the maturation of the nervous system^34^. Therefore, the observed alterations might have long-term consequences on the survival and fitness of hosts^16^.

### Environmental factors associated with changes in the urban gut microbiota

One of the primary goals of this study was to determine whether distinct environmental variables characterising the urban space can predict changes in the gut microbiota of great tit nestlings. Based on our models, tree cover density was the only environmental axis that covaried with alpha diversity (whilst also confirming that alpha diversity was not spatially or temporally autocorrelated). In line with our findings, white-crowned sparrows occupying territories with greater tree cover also exhibited more diverse gut microbial communities than those occupying regions with less tree cover^14,15^. This is likely to be explained by the tree cover density influencing the diversity and abundance of invertebrates^35^, which are the primary food source for great tits during the chick-rearing period^36^. Reductions in tree cover density, often observed in urban spaces, can also direct animals to search for anthropogenic food^37^. Consistently, the camera recordings of the nests sampled in this study revealed that the amount of anthropogenic food brought into the nests was higher in territories characterized by high ISA percentages than in low-ISA territories(Corsini et al., in prep.), and dietary alterations are known to be associated with changes in alpha diversity^29,38,39^. However, our predictions regarding their diets remain speculative, as we did not analyse the diets of the studied nestlings. Furthermore, it is important to note that tree cover density is correlated with all other variables used in the study (Fig. 3b, Supplementary Table 3). Specifically, distance to the closest road, NDVI, light and sound pollution had relatively strong associations with tree cover density. Although these variables were not retained in the final models due to multicollinearity issues, the observed alpha diversity patterns might stem from underlying relationships between these variables.

An important aim of the study was to determine the environmental parameters predicting compositional changes in gut microbiota. Although seasonal fluctuations can influence gut microbiota^40^, we did not find evidence that temporal variation in sampling leads to compositional changes. However, microbial community composition exhibited spatial structuring. One potential explanation is that the microbial communities residing in the guts of studied nestlings, at least to some extent, represent the environmental pool of microorganisms^41^, which exhibits microgeographic variations^42^. Alternatively, these changes might be related to spatial variation in environmental variables. Based on our models, the two variables that showed the strongest associations with change in the microbial community composition were sound pollution and the distance to the city centre. Sound pollution can influence gut microbiota by different but non-exclusive mechanisms. Noise might impair parent–offspring communication by masking the begging calls of the young, and parents might adjust their food provisioning accordingly^43^. Therefore, the amount of food received by the young might vary depending on the background noise levels, which in turn could influence gut microbiota. Alternatively, acoustic stress can activate the hypothalamic–pituitary–adrenal axis and alter the endocrine profiles^9,44^, causing cascading impacts on glucose metabolism^45^, immune function^46^, oxidative stress^47^ and gut physiology^48^ and consequently influencing gut microbiota^45,48^. The second strongest predictive variable was the distance to the city center. This interaction might be mediated by environmental variables distributed linearly from the city centre such as light pollution. Indeed, light pollution was a significant predictor based on most of our models and can indirectly affect gut microbiota by altering the host physiology^49^. Alternatively, the observed correlations between the distance to the city center and community composition might be associated through unmeasured chemical pollutants showing collinearity with distance from the city centre, such as particulate matter in ambient air or trace metals^50^. Indeed, environmental chemical exposures have been shown to elicit negative effects on avian physiology^51^ and consequently can influence gut microbiota^52^. It is important to note that due to the multicollinearity among these variables, it is not possible to infer the exact correlative factors explaining changes in the microbial community composition, and each of these assumptions requires further experimental testing to infer the exact causative factors driving the observed differences in beta diversity.

## Conclusions and outlook

We quantified the extent to which anthropogenic modifications pertaining to the urban environment -including the use of artificial nestboxes- reshapes animal-microbe interactions in great tit nestlings. We report clear trends regarding urban-related shifts in taxonomic composition, a reduction in alpha diversity, and increased pathogen susceptibility. While the exact causal interactions between the environmental parameters and observed microbial changes remain to be tested experimentally, we outlined different mechanisms that are likely to be the driving forces of the measured microbiota variation in the studied urban space. This study thus fills an important gap in providing pioneering evidence on how multi-dimensional anthropogenic changes may relate to gut microbiota during early nestling development, the most critical life-history stage during which gut microbial communities are established and program several developmental processes of their hosts^16,34,53^. Another novel piece of information provided by our study is how the use of nestboxes, the golden standard of ecological field studies, can affect the outcome of research investigating gut microbial communities, an important point that should be considered in future investigations. Our study further adds to the existing evidence that how the urban space is defined might have a prominent impact on the outcome of ecological studies.

Although our study shed light on how anthropogenic change can influence gut microbiota assembly in early life, it also has some potential limitations. First, our relatively small sample size did not allow us to use more sophisticated analysis methods such as machine learning implications to gain deeper insights into drivers of microbial change in urban space. Another drawback regarding the sample size is that our conclusions on cavity type’s influence on gut microbiota rely on a small dataset. Consequently, these preliminary findings should be interpreted with caution until they are confirmed by larger datasets. Second, our sampling design was somehow unbalanced, with a larger number of samples collected from urban sites. Although unlikely, we cannot rule out the possible effect of unequal sample size on study outcomes. Third, our sampling was not replicated spatially and temporally; ultimately, the results presented in this work should be assessed by future studies conducted in multiple cities and across multiple years.

Importantly, our study provides important evidence that highlights future opportunities and further refinement in research questions. First, it is pivotal to conduct experimental testing in which the confounding variables are strictly controlled to infer the exact causal effects of the identified environmental factors covarying with microbial changes. Second, whether and how gut microbes can facilitate the adaptation of their hosts to changing environments is an important point that remains to be investigated to achieve full comprehension of the long-term fitness consequences of host-microbe interactions. Third, our study raises the possibility of answering a central question in microbial ecology by leveraging the urban–rural gradient; cross-fostering experiments among contrasting habitats would allow us to disentangle the relative importance of host-specific and environmental factors in shaping microbial communities. Answering these questions would allow us to fully comprehend the ecological and evolutionary dynamics and consequences of animal-microbe symbiosis, in the context of globally increasing urbanisation, specifically in the fields of urban planning, wildlife and public health management.

## Materials and methods

### Ethics declarations

The experimental protocols, including handling the birds, were approved by the Regional Directorate for Environmental Protection (RDOŚ) in Warsaw, Poland. All experiments were carried out in accordance with relevant guidelines and regulations.

### Study sites

Great tits are hole-nesting passerine birds known to occupy a wide range of habitats, from primary forests^54^ to urban city centres^55^. In this species, the nesting environment is an important driver that shapes gut microbiota during early development^56^, making the great tit an ideal subject to study microbial changes across an urban mosaic. Here, we monitored the great tit reproductive cycle in the capital city of Warsaw in Poland, where 565 woodcrete Schwegler nestboxes (type 1b) were set up at nine sites located within and outside the city (Fig. 1; Supplementary Table 5). We included the following sampling sites: a natural forest (c. 10 km from the city borders and approximately 20 km from the city centre), a peri-urban village bordering a natural forest, two residential areas, two urban woodlands, a large urban park and an office area (2019, 2020). Furthermore, natural cavities located on a Primeval Forest within the urban administrative border were also monitored (Fig. 4).

### Capturing urbanisation

The urban space was defined with two different frameworks: under the first framework, each nest was categorised depending on whether it was located within the administrative limits of the city (urban) or outside of these limits (rural). Based on this dichotomous categorization, the urban sites included an office area, two residential areas, an urban forest, an urban park and two urban woodlands. The rural sites comprised a natural forest and a peri-urban village (Supplementary Table 5). Under the second framework, we aimed to capture the heterogeneity in actual surface use patterns and defined urbanisation using the percentage of impervious surface area (ISA) surrounding the respective location of every nest analysed in the study. A map of ISA in Warsaw (in a range from 0 to 100%) with a 20-m pixel resolution was downloaded from Copernicus Land Monitoring Services. ISA included all built-up areas and soil-sealing surfaces substituting original or semi-natural surfaces. Averaged ISA values at each nest were obtained using a 100-m-radius buffer starting from each nestbox location and using geographic information system tools (GIS). The radius was selected on literature-based estimates of parental foraging: blue tits travel about 53.2 m (22.9 SD) in food-poor (but natural) environments while feeding the nestlings^57^; importantly, the same study has shown that birds also flied beyond 50 m from the nest location in c.1/3 of their foraging trips^57^, therefore, a 100 m radius around each nestbox constitutes a conservative estimate of food foraging distances covered by parents while feeding the nestlings. ISA percentage was used as a continuous variable in alpha and beta diversity analyses.

### Environmental and spatial variables

We collected ten environmental and spatial variables as described previously^1,58,59^ (also see Supplementary Text 1 for details). Regarding the environmental variables collected on the ground, the (1) *human presence* was derived by quantifying all humans and dogs, with repeated 30-s long counts performed within a 15-m radius around each nestbox (as detailed in Corsini et al.^59^). (2) *Sound pollution* was obtained after averaging recordings that occurred on the DbC scale using hand-held sound level meters equipped with a microphone, over four days throughout the field season, three times per day. (3) *Temperature* was obtained from 22 Thermocrones ibuttons DS1921G set from 24/04/18 until 30/06/18with a 1-h sampling frequency, and distributed across the entire gradient of urbanisation, following Szulkin et al.^1^.

Spatial variables such as the distances from each nestbox to (4) the *closest road* and to (5) the *closest path *(*for vehicular and pedestrian use, respectively*)* and* (6) the *city centre* (i.e., the location of the Palace of Culture and Sciences) were computed and measured in metres using the “Measure line” tool in QGIS, as described by Corsini et al.^59^. Furthermore, (7) *a distance matrix of the geographical coordinates* of the sampling sites was calculated based on the Haversine distances.

We also extracted environmental variables using digital photography and satellite imagery techniques and computed the values of these variables at the nest level in a 100-m-radius buffer. For (8) *light pollution*, a map of light pollution in Warsaw with a 10-m pixel resolution was extrapolated from night-time digital photographic images shot on 08/10/2015 by astronauts from the International Space Station^60^. For (9) *tree cover density*, a map of tree cover density in Warsaw (in a range from 0 to 100%) with a 20-m pixel resolution was downloaded from Copernicus Land Monitoring Services. For a proxy of live green vegetation, (10) the normalized difference vegetation index (*NDVI*) was estimated using satellite images derived from SENTINEL2 that are available on the Earth Explorer website (https://earthexplorer.usgs.gov).

### Sample collection, DNA extraction and library preparation

Faecal samples were collected from 15-day-old great tit chicks hatched in 89 nests (eight and 81 nests were located in natural cavities and nestboxes, respectively; Supplementary Table 5), between the 19th of May 2018 and the 20th of July 2018. Faeces of one chick per nest (from the largest chick in the brood) was collected, except for four nests where more than one chick per nest was sampled (all originating from woodcrete nestboxes). The samples were collected noninvasively while handling the chicks and directly deposited in 5-mL sterile Eppendorf tubes, each filled with 3 mL of RNAlater (Qiagen) and further stored at − 20 °C.

Details of the microbial analysis are described in Maraci et al.^61^. In short, microbial DNA was extracted using the QIAamp PowerFecal DNA Kit (Qiagen), as described in the manufacturer’s protocol. The hypervariable V3–V4 region of the 16S ribosomal RNA (rRNA) gene was targeted following the Illumina 16S Metagenomic Library Preparation Guide, 15044223-B. The final amplicon pool contained a pool of blank controls for DNA extraction and PCR amplification and one replicate of a single sample, alongside 94 biological samples sequenced on the Illumina MiSeq system (Illumina, Inc., San Diego, CA, USA).

### Bioinformatic processing

The bioinformatic processing was carried out as described in detail^62^. In short, MiSeq PE reads were assembled in an iterative manner using Flash v1.2.11^63^. All other bioinformatic steps consisted of (1) adapter clipping with cutadapt v1.18^64^; (2) de-replication, alignment, filtering and de-noising with mothur v1.41.3^65^; (3) chimaera checking and operational taxonomic unit (OTU) clustering with USEARCH v8.0.1477^66^; and (4) taxonomic classification based on the full SILVA database v132^67^.

### Statistical analyses

All consecutive statistical analyses were conducted in R version 4.0.0^68^ and Primer-e software version 7^69^. As an initial quality filtering step, all OTUs that could not be classified at the phylum level or that were classified as mitochondria or chloroplasts were discarded. Next, all OTUs that were not represented by at least one read in 2% of the samples or with fewer read counts than 0.001% of the total number of reads were excluded. Subsequently, samples with a lower read count than 900 (N = 12) were removed from the dataset. We evaluated whether our sample size is sufficient for an accurate estimation using OTU accumulation curve.

To test whether the microbial communities were affected by cavity type, we created a subset of data including samples collected from nestboxes (N = 7) and those collected from natural cavities (N = 6) located in the same urban forest, a highly homogenous ecological site (see^18^, for a map for the distribution of the natural cavities and nestboxes across the study site). Subsequently, the six samples obtained from natural cavities were excluded from the remaining analyses, and the dataset used in further analyses ultimately consisted of 76 samples collected from nestboxes (Supplementary Table 5). Using a two-step approach, we analysed the interactions among microbial community differences, urbanisation and environmental change. First, the urban space was defined using two different frameworks based on (1) the often-used administrative border delineation (urban/rural site) and (2) the percentage of impervious surface area (ISA) surrounding each nest, irrespective of the location of the nest within or outside of the city borders. Thus, microbial alpha and beta diversity was compared (1) between urban and rural sites, and also (2) examined in relation to ISA percentage.

Before the alpha diversity analyses, we rarefied OTU read count data to the lowest read count observed in the dataset (937). Then, we calculated Shannon’s diversity index, which accounts for both the abundance and evenness of the taxa present^70^, the number of observed OTUs, which estimates species richness, and Faith's phylogenetic diversity, which incorporates phylogenetic relationships between microbial taxa^71^, as the microbial alpha diversity estimates. We normalised these indexes employing square root transformation. We examined whether these alpha diversity indexes differed between nestboxes and natural cavities by fitting linear models, implemented using the *lm* function of R package stats^68^.To analyse the impact of urbanisation on alpha diversity, we employed different linear models (LMs) using square-root-transformed Shannon’s diversity, the number of observed OTUs, or Faith’s phylogenetic diversity indices of the samples collected from nestboxes as the response variable. Depending on the framework used to define urbanisation, we used the urban/rural categorisation or ISA (as a continuous variable) as the fixed effect. Subsequently, to account for the potential impact of differences in the sample collection dates on alpha diversity, we fitted LMs using the number of days between initiation of the study and sample collection as a fixed effect, and Shannon’s diversity index, the number of observed OTUs or Faith’s phylogenetic diversity as the response variable. The associations between distinct environmental and spatial variables and microbial alpha diversity were also inferred. As an initial step, the extent to which the environmental parameters varied between urban and rural sites was tested with Welch's two-sample t-test. Correlations between alpha diversity, ISA and other environmental variables were then investigated using Pearson’s correlation. To analyse the interactions between alpha diversity and environmental variables, three linear mixed models (LMMs) were fitted using Shannon’s diversity, the number of observed OTUs, or Faith’s phylogenetic diversity indices as the response variables and all environmental variables as fixed effects, as implemented using lme4 version 1.1-25^72^. To examine multicollinearity between the variables, the variance inflation factor (VIF) was calculated with the *Car* package^73^, and the predictors were sequentially removed from the model based on VIF values and biological relevance, and VIF values were recalculated^74^. This procedure was repeated until all the VIF values were smaller than two. The final models were fitted with the remaining environmental variables as fixed effects, that is: distance to the city centre, human presence, temperature, tree cover density and distance to the closest path. The sampling site was included in these LMMs as random effects to account for the nonindependence of the samples originating from the same sampling site. Furthermore, we also tested for spatial autocorrelation in the simulated scaled residuals of the fitted LMs and LLMs by Moran’s I test as implemented in the *DHARMa* package^75^.

The taxonomic and compositional structures of the microbial communities collected from different localities were visualized with stacked bar plots based on family-level taxonomy using *ggplot2* version 3.3.2^76^. To identify differentially abundant OTUs in samples collected from urban and rural localities, the logarithmic fold changes between groups were estimated using a negative binomial Wald test implemented in *Deseq2* version 1.12.4 extension^22^ of the *Phyloseq* package version 1.32.0^77^. The significance threshold of the *p* values was set as 0.05 after a Benjamini and Hochberg false‐discovery rate correction^78^.

To analyse community composition^79^, first, the filtered absolute abundance data were normalized by applying cumulative sum scaling (CSS) normalization in the R package *Metagenomeseq* version 1.30.0^80^ to account for unequal sequence coverage. The differences in community composition between urban and rural sites were visualized using non-metric multidimensional scaling (nMDS) based on Bray–Curtis^81^, and the weighted UniFrac^82^ dissimilarities implemented using the *Vegan* package version 2.5^83^. We examined microbial composition in relation to whether samples originated from urban or rural areas, sampling site, ISA percentage (as a continuous value) and sampling time using permutational multivariate analyses of variance (PERMANOVA^84^, with Bray–Curtis and the weighted UniFrac dissimilarities, implemented in Primer-e software with 9999 permutations. Subsequently, using the same dissimilarity measures, we examined the effect of cavity type on microbiota composition by PERMANOVA. We also tested homogeneity of group dispersions using PERMDISP in Primer-e. Finally, the interactions between spatial and environmental variables and beta diversity were also evaluated by employing different methods. First, to assess the spatial autocorrelation of beta diversity, we tested the correlations between the Bray–Curtis and weighted UniFrac dissimilarity matrices and the distance matrix of the geographical coordinates of the sampling sites, using the mantel test. Second, we employed a partial mantel test to investigate the correlations between Bray–Curtis and weighted UniFrac microbial dissimilarity matrices and the distance matrices of the environmental variables obtained based on Euclidean distances, controlling for the effect of the distance matrix of the geographical coordinates of the sampling sites. Third, to identify the variables that were strongly related to the first two ordination axes, the multiple regression between each environmental variable and the ordination axes was evaluated using the *Envfit* function, and the significance of each correlation was tested based on 9999 permutations. Finally, the subset of environmental variables whose Euclidean distance matrices correlated maximally with the microbial distance matrix based on Bray–Curtis was identified employing the *BIOENV* procedure^85^.

## Supplementary Information

Supplementary Information.

## Data Availability

Sequences generated for this study have been uploaded to the European Nucleotide Archive (ENA) repository under the accession number: PRJEB44290. The scripts used for processing the data are provided in the GitHub repository at: https://github.com/AnnaAntonatouPap/Urban-related-changes-in-tit-microbiota.

## References

[1] Szulkin M (2020). How to quantify urbanization when testing for urban evolution?. Urban Evol. Biol..

[2] Slabbekoorn H (2013). Songs of the city: Noise-dependent spectral plasticity in the acoustic phenotype of urban birds. Anim. Behav..

[3] Christiansen NA, Fryirs KA, Green TJ, Hose GC (2019). The impact of urbanisation on community structure, gene abundance and transcription rates of microbes in upland swamps of Eastern Australia. PLoS ONE.

[4] Alberti M (2017). Global urban signatures of phenotypic change in animal and plant populations. Proc. Natl. Acad. Sci. USA.

[5] McFall-Ngai MM (2013). Animals in a bacterial world, a new imperative for the life sciences. Proc. Natl. Acad. Sci..

[6] Zilber-Rosenberg I, Rosenberg E (2008). Role of microorganisms in the evolution of animals and plants: the hologenome theory of evolution. FEMS Microbiol. Rev..

[7] Trevelline BK, Fontaine SS, Hartup BK, Kohl KD (2019). Conservation biology needs a microbial renaissance: A call for the consideration of host-associated microbiota in wildlife management practices. Proc. R. Soc. B Biol. Sci..

[8] Jarrett C, Powell LL, McDevitt H, Helm B, Welch AJ (2020). Bitter fruits of hard labour: diet metabarcoding and telemetry reveal that urban songbirds travel further for lower-quality food. Oecologia.

[9] Zollinger SA (2019). Traffic noise exposure depresses plasma corticosterone and delays offspring growth in breeding zebra finches. Conserv. Physiol..

[10] Sprau P, Mouchet A, Dingemanse NJ (2017). Multidimensional environmental predictors of variation in avian forest and city life histories. Behav. Ecol..

[11] Teyssier A (2018). Inside the guts of the city: Urban-induced alterations of the gut microbiota in a wild passerine. Sci. Total Environ..

[12] Murray MH (2020). Gut microbiome shifts with urbanization and potentially facilitates a zoonotic pathogen in a wading bird. PLoS ONE.

[13] Fuirst M, Veit RR, Hahn M, Dheilly N, Thorne LH (2018). Effects of urbanization on the foraging ecology and microbiota of the generalist seabird *Larus argentatus*. PLoS ONE.

[14] Phillips JN, Berlow M, Derryberry EP (2018). The effects of landscape urbanization on the gut microbiome: An exploration into the gut of urban and rural white-crowned sparrows. Front. Ecol. Evol..

[15] Berlow M, Phillips JN, Derryberry EP (2020). Effects of urbanization and landscape on gut microbiomes in white-crowned sparrows. Microb. Ecol..

[16] Cox LM (2014). Altering the intestinal microbiota during a critical developmental window has lasting metabolic consequences. Cell.

[17] Knutie SA, Wilkinson CL, Kohl KD, Rohr JR (2017). Early-life disruption of amphibian microbiota decreases later-life resistance to parasites. Nat. Commun..

[18] Sudyka, J., Di Lecce, I., Wojas, L., Rowiński, P. & Szulkin, M. Nest-boxes alter the reproductive ecology of urban cavity-nesters in a species-dependent way. 10.32942/OSF.IO/WP9MN.

[19] Maziarz M, Broughton RK, Wesołowski T (2017). Microclimate in tree cavities and nest-boxes: Implications for hole-nesting birds. For. Ecol. Manag..

[20] Thompson MJ, Capilla-Lasheras P, Dominoni DM, Réale D, Charmantier A (2022). Phenotypic variation in urban environments: mechanisms and implications. Trends Ecol. Evol..

[21] Salmón P (2021). Continent-wide genomic signatures of adaptation to urbanisation in a songbird across Europe. Nat. Commun..

[22] Love MI, Huber W, Anders S (2014). Moderated estimation of fold change and dispersion for RNA-seq data with DESeq2. Genome Biol..

[23] Sackey BA, Mensah P, Collison E, Sakyi-Dawson E (2001). Campylobacter, Salmonella, Shigella and *Escherichia coli* in live and dressed poultry from metropolitan Accra. Int. J. Food Microbiol..

[24] Benskin CMWH, Wilson K, Jones K, Hartley IR (2009). Bacterial pathogens in wild birds: A review of the frequency and effects of infection. Biol. Rev..

[25] Hansell M, Overhill R (2000). Bird nests and construction behaviour. Bird Nests Constr. Behav..

[26] Siddiqui SH, Khan M, Kang D, Choi HW, Shim K (2022). Meta-analysis and systematic review of the thermal stress response: *Gallus gallus* domesticus show low immune responses during heat stress. Front. Physiol..

[27] Sepulveda J, Moeller AH (2020). The effects of temperature on animal gut microbiomes. Front. Microbiol..

[28] Kohl KD, Yahn J (2016). Effects of environmental temperature on the gut microbial communities of tadpoles. Environ. Microbiol..

[29] Teyssier A (2020). Diet contributes to urban-induced alterations in gut microbiota: Experimental evidence from a wild passerine. Proc. R. Soc. B Biol. Sci..

[30] Benskin CMWH, Rhodes G, Pickup RW, Wilson K, Hartley IR (2010). Diversity and temporal stability of bacterial communities in a model passerine bird, the zebra finch. Mol. Ecol..

[31] Garrett WS (2010). Enterobacteriaceae Act in concert with the gut microbiota to induce spontaneous and maternally transmitted colitis. Cell Host Microbe.

[32] Videvall E (2020). Early-life gut dysbiosis linked to juvenile mortality in ostriches. BMC Microbiome.

[33] Hooper LV, MacPherson AJ (2010). Immune adaptations that maintain homeostasis with the intestinal microbiota. Nat. Rev. Immunol..

[34] Borre YE (2014). Microbiota and neurodevelopmental windows: Implications for brain disorders. Trends Mol. Med..

[35] Jones EL, Leather SR (2012). Invertebrates in urban areas: A review. Eur. J. Entomol..

[36] Wilkin TA, King LE, Sheldon BC (2009). Habitat quality, nestling diet, and provisioning behaviour in great tits Parus major. J. Avian Biol..

[37] Pollock CJ, Capilla-Lasheras P, McGill RAR, Helm B, Dominoni DM (2017). Integrated behavioural and stable isotope data reveal altered diet linked to low breeding success in urban-dwelling blue tits (*Cyanistes caeruleus*). Sci. Rep..

[38] Davidson GL (2020). Diet induces parallel changes to the gut microbiota and problem solving performance in a wild bird. Sci. Rep..

[39] Bodawatta KH (2021). Flexibility and resilience of great tit (*Parus major*) gut microbiomes to changing diets. Anim. Microbiome.

[40] Baniel A (2021). Seasonal shifts in the gut microbiome indicate plastic responses to diet in wild geladas. Microbiome.

[41] Sullam KE (2012). Environmental and ecological factors that shape the gut bacterial communities of fish: A meta-analysis. Mol. Ecol..

[42] Martiny JBH (2006). Microbial biogeography: Putting microorganisms on the map. Nat. Rev. Microbiol..

[43] Lucass C, Eens M, Müller W (2016). When ambient noise impairs parent-offspring communication. Environ. Pollut..

[44] Kight CR, Swaddle JP (2011). How and why environmental noise impacts animals: An integrative, mechanistic review. Ecol. Lett..

[45] Cui B, Gai Z, She X, Wang R, Xi Z (2016). Effects of chronic noise on glucose metabolism and gut microbiota-host inflammatory homeostasis in rats. Sci. Rep..

[46] Campo JL, Gil MG, Dávila SG (2005). Effects of specific noise and music stimuli on stress and fear levels of laying hens of several breeds. Appl. Anim. Behav. Sci..

[47] Injaian AS, Taff CC, Patricelli GL (2018). Experimental anthropogenic noise impacts avian parental behaviour, nestling growth and nestling oxidative stress. Anim. Behav..

[48] Cui B (2018). Effects of chronic noise exposure on the microbiome-gut-brain axis in senescence-accelerated prone mice: Implications for Alzheimer’s disease. J. Neuroinflammation.

[49] Wei L (2020). Constant light exposure alters gut microbiota and promotes the progression of steatohepatitis in high fat diet rats. Front. Microbiol..

[50] Chatelain M (2021). Replicated, urban-driven exposure to metallic trace elements in two passerines. Sci. Rep..

[51] Chatelain M (2021). Urban metal pollution explains variation in reproductive outputs in great tits and blue tits. Sci. Total Environ..

[52] Rosenfeld CS (2017). Gut dysbiosis in animals due to environmental chemical exposures. Front. Cell. Infect. Microbiol..

[53] Sommer F, Bäckhed F (2013). The gut microbiota-masters of host development and physiology. Nat. Rev. Microbiol..

[54] Tomiałojć L, Wesołowski T (2004). Diversity of the Białowieza forest avifauna in space and time. J. Ornithol..

[55] Corsini M (2021). Growing in the city: Urban evolutionary ecology of avian growth rates. Evol. Appl..

[56] Teyssier A, Lens L, Matthysen E, White J (2018). Dynamics of gut microbiota diversity during the early development of an avian host: Evidence from a cross-foster experiment. Front. Microbiol..

[57] Tremblay I, Thomas D, Blondel J, Perret P, Lambrechts MM (2005). The effect of habitat quality on foraging patterns, provisioning rate and nestling growth in Corsican Blue Tits Parus caeruleus. Ibis (Lond 1859)..

[58] Corsini M, Marrot P, Szulkin M (2019). Quantifying human presence in a heterogeneous urban landscape. Behav. Ecol..

[59] Corsini M, Dubiec A, Marrot P, Szulkin M (2017). Humans and tits in the city: Quantifying the effects of human presence on great tit and blue tit reproductive trait variation. Front. Ecol. Evol..

[60] Kyba CCM (2015). High-resolution imagery of earth at night: New sources, opportunities and challenges. Remote Sens..

[61] Maraci Ö (2021). The gut microbial composition is species-specific and individual-specific in two species of estrildid finches, the Bengalese finch and the zebra finch. Front. Microbiol..

[62] Engel K (2017). Individual- and species-specific skin microbiomes in three different estrildid finch species revealed by 16S amplicon sequencing. Microb. Ecol..

[63] Magoč T, Salzberg SL (2011). FLASH: Fast length adjustment of short reads to improve genome assemblies. Bioinformatics.

[64] Martin M (2011). Cutadapt removes adapter sequences from high-throughput sequencing reads. EMBnet.journal.

[65] Schloss PD (2009). Introducing mothur: Open-source, platform-independent, community-supported software for describing and comparing microbial communities. Appl. Environ. Microbiol..

[66] Edgar RC (2010). Search and clustering orders of magnitude faster than BLAST. Bioinformatics.

[67] Quast C (2013). The SILVA ribosomal RNA gene database project: Improved data processing and web-based tools. Nucleic Acids Res..

[68] R Core Team (2020). R: A language and environment for statistical computing.

[69] Clarke, K. R., Gorley, R., Somerfield, P. & Warwick, R. *Change in Marine Communities: an Approach to Statistical Analysis and Interpretation* 3rd edn (Prim. Plymouth, 2014).

[70] Shannon CE (1997). The mathematical theory of communication. MD Comput..

[71] Faith DP (1992). Conservation evaluation and phylogenetic diversity. Biol. Conserv..

[72] Bates D, Mächler M, Bolker BM, Walker SC (2015). Fitting linear mixed-effects models using lme4. J. Stat. Softw..

[73] Fox, J. *et al.* The car Package. *R* (2012).

[74] Zuur AF, Ieno EN, Elphick CS (2010). A protocol for data exploration to avoid common statistical problems. Methods Ecol. Evol..

[75] DHARMa: Residual diagnostics for hierarchical (multi-level/mixed) regression models. https://cran.r-project.org/web/packages/DHARMa/vignettes/DHARMa.html.

[76] Wickham H (2009). ggplot2: Elegant Graphics for Data Analysis.

[77] McMurdie PJ, Holmes S (2013). Phyloseq: An R package for reproducible interactive analysis and graphics of microbiome census data. PLoS ONE.

[78] Benjamini Y, Hochberg Y (1995). Controlling the false discovery rate: A practical and powerful approach to multiple testing. J. R. Stat. Soc. Ser. B.

[79] Whittaker RH (1960). Vegetation of the Siskiyou mountains Oregon and California. Ecol. Monogr..

[80] Paulson, J. metagenomeSeq: Statistical analysis for sparse high-throughput sequencing. *Bioconductor.Jp* (2014).

[81] Bray JR, Curtis JT (1957). An ordination of the upland forest communities of southern Wisconsin. Ecol. Monogr..

[82] Lozupone CA, Hamady M, Kelley ST, Knight R (2007). Quantitative and qualitative β diversity measures lead to different insights into factors that structure microbial communities. Appl. Environ. Microbiol..

[83] Oksanen, J. *et al. Package ‘vegan’ Title Community Ecology Package Version 2.5-6*. *cran.ism.ac.jp* (2019).

[84] Anderson MJ, Anderson MJ (2001). A new method for non-parametric multivariate analysis of variance. Austral Ecol..

[85] Clarke KR, Ainsworth M (1993). A method of linking multivariate community structure to environmental variables. Mar. Ecol. Prog. Ser..

[86] QGIS Development Team (2019). QGIS Geographic Information System.

