# Supplementary material for: Changes to the gut microbiota of a wild juvenile passerine in a multidimensional urban mosaic
Source: Sci Rep. 2022 Apr 27;12:6872. doi: 10.1038/s41598-022-10734-7 (PMC9046431; doi:10.1038/s41598-022-10734-7)
Supplement: Supplementary file 1 — Supplementary Information. [file 41598_2022_10734_MOESM1_ESM.pdf]

Supplemental Information for:

Changes to the Gut Microbiota of a Wild Juvenile Passerine in a Multidimensional Urban Mosaic

Öncü Maraci, Michela Corsini, Anna Antonatou-Papaioannou, Sebastian Jünemann,

Joanna Sudyka, Irene Di Lecce, Barbara A. Caspers, Marta Szulkin

Table of Contents:

**Supplementary Figure 1**	Page 2
**Supplementary Table 1**	Page 3
**Supplementary Table 2**	Page 4
**Supplementary Table 3**	Page 5
**Supplementary Table 4**	Page 6
**Supplementary Table 5**	Page 7
**Supplementary Table 6**	Page 8
**Supplementary Table 7**	Page 9
**Supplementary Text 1**	Page10

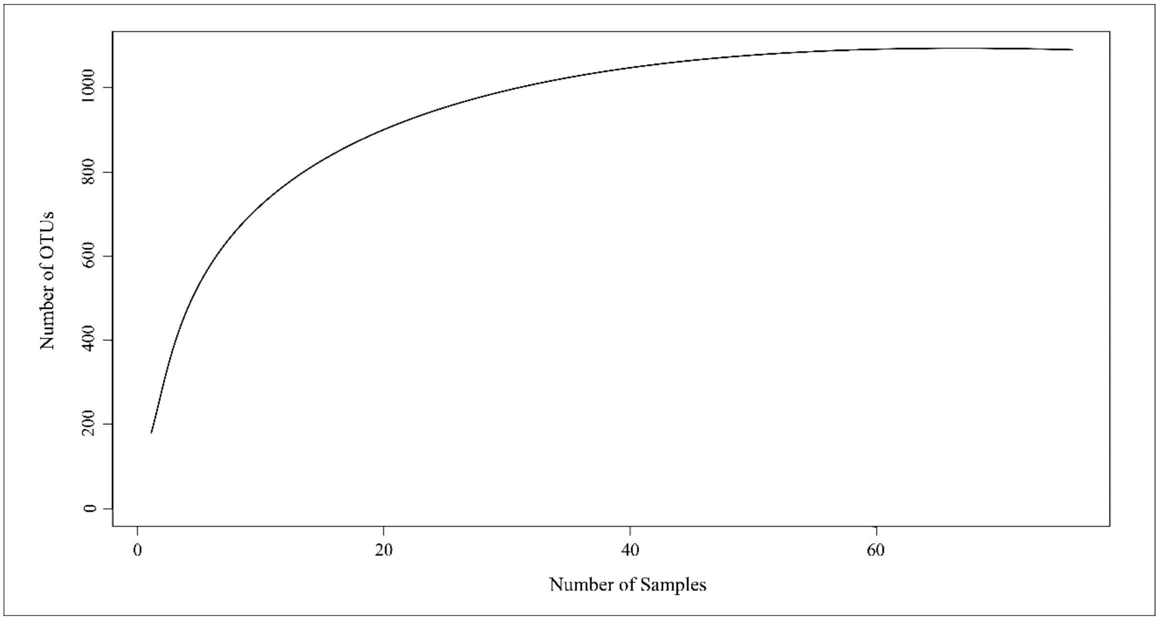

Supplementary Figure 1. OTU accumulation curve with sample size.

Supplementary Table 1. Linear models and Moran I test.

Linear Models	Response variable	N	Est	SE	CI		p	Moran I p
Cavity type/alpha diversity	**Shannon**	**13**	**Natural cavity: -0.60**	**0.22**	**-1.09**	**0.11**	**0.021**	NA
	No of OTUs	13	Natural cavity: -3.10	1.44	-6.28	0.08	0.055	NA
	Faith's PD	13	Natural cavity: -1.25	0.73	-2.87	0.36	0.115	NA
Sampling collection day/alpha diversity	Shannon	76	0	0	-0.01	0.01	0.956	NA
	No of OTUs	76	-0.03	0.02	-0.07	0.01	0.088	NA
	Faith's PD	76	0.01	0.01	-0.01	0.03	0.446	NA
Urban-rural/alpha diversity	Shannon	76	Urban: -0.22	0.12	-0.46	0.03	0.084	0.19
	No of OTUs	76	Urban: -1.22	0.8	-2.81	0.37	0.131	0.34
	**Faith's PD**	**76**	**Urban: -0.72**	**0.36**	**-1.44**	**-0.01**	**0.048**	0.22
ISA/alpha diversity	**Shannon**	**76**	**-0.01**	**0**	**-0.02**	**0**	**0.019**	0.31
	**No of OTUs**	**76**	**-0.08**	**0.03**	**-0.13**	**-0.03**	**0.004**	0.94
	**Faith's PD**	**76**	**-0.04**	**0.01**	**-0.06**	**-0.02**	**0.001**	0.82

Supplementary Table 2. The distribution of ISA in urban and rural sites.

ISA	Rural	Urban
**N**	25	57
**Min**	0	0
**Max**	13.61	59.20
**Mean**	2.40	13.11
**Std. error**	0.82	1.99
**Stand. dev**	4.12	15.08
**Median**	0	6.25

Supplementary Table 3. Pearson's correlation coefficients among ISA and environmental variables.

	ISA	City center	Light	Temperature	Human	NDVI	Sound	Closest road	Closest path	Tree cover
ISA	NA	**-0.43**	**0.87**	**0.3**	**0.48**	**-0.8**	**0.51**	**-0.44**	**-0.23**	**-0.6**
City center		NA	**-0.58**	0.12	**-0.39**	-0.06	**-0.67**	**0.53**	0.2	**0.26**
Light			NA	0.2	**0.57**	**-0.59**	**0.53**	**-0.4**	-0.18	**-0.44**
Temperature				NA	**0.25**	**-0.52**	**0.27**	**-0.25**	-0.1	**-0.51**
Human					NA	**-0.41**	**0.41**	**-0.31**	-0.15	**-0.4**
NDVI						NA	-0.15	0.09	0.08	**0.52**
Sound							NA	**-0.77**	**-0.37**	**-0.66**
Closest road								**NA**	**0.55**	**0.78**
Closest path									**NA**	**0.47**
Tree cover										**NA**

Supplementary Table 4. Differentially abundant OTUs between urban and rural samples.

OTU	Abundant	change	lfcSE	padj	Family	Genus
2	Urban	6.71	1.07	0.000	*Catellicoccaceae*	*Catellicoccus*
6	Rural	-3.21	0.80	0.002	*Enterococcaceae*	*Enterococcus*
7	Urban	3.89	1.20	0.025	*Enterobacteriaceae*	*Escherichia-Shigella*
16	Urban	2.19	0.65	0.021	*Micrococcaceae*	*Pseudarthrobacter*
26	Urban	2.84	0.88	0.025	*Pseudonocardiaceae*	*Pseudonocardia*
27	Rural	-2.70	0.89	0.040	*Peptostreptococcaceae*	*Peptostreptococcaceae_unclassified*
36	Rural	-2.35	0.56	0.002	*Xanthobacteraceae*	*Xanthobacteraceae_unclassified*
49	Urban	2.97	0.98	0.040	*Carnobacteriaceae*	*Carnobacterium*
60	Rural	-3.80	0.94	0.002	*Beijerinckiaceae*	*Roseiarcus*
63	Rural	-4.65	1.06	0.001	*Acidothermaceae*	*Acidothermus*
90	Rural	-4.50	0.97	0.000	*Solirubrobacteraceae*	*Conexibacter*
106	Urban	3.38	0.77	0.001	*Sphingomonadaceae*	*Sphingomonas*
108	Urban	2.59	0.78	0.023	*Microbacteriaceae*	*Agromyces*
118	Rural	-3.54	0.84	0.001	*Mycobacteriaceae*	*Mycobacterium*
119	Rural	-4.68	1.48	0.028	*Acidothermaceae*	*Acidothermus*
149	Rural	-5.65	1.42	0.002	*Mycobacteriaceae*	*Mycobacterium*
186	Urban	2.99	0.92	0.025	*Iamiaceae*	*Iamia*
196	Urban	2.32	0.77	0.040	*Devosiaceae*	*Devosia*
243	Rural	-3.33	1.04	0.026	*Xanthobacteraceae*	*uncultured*
304	Urban	2.93	0.88	0.023	*MB-A2-108_fa*	*MB-A2-108_ge*

Supplementary Table 5. Differences in environmental and spatial variables between rural and urban sites based on Welch's two-sample t-test.

Urban/Rural							
**Variable**	**t**	**df**	**p**	**95% confidence interval**		**Mean Urban**	**Mean Rural**
Distance to the city center	55.27	68.68	**<0.001**	17.19	18.47	3.37	21.20
Light pollution	-7.85	50.31	**<0.001**	-6846.61	-4057.49	6507.87	1055.82
Sound pollution	-5.90	27.87	**<0.001**	-20.16	-9.78	63.32	48.35
Human presence	-4.69	55.45	**<0.001**	-1.28	-0.51	1.03	0.14
Distance to the closest road	4.11	24.81	**<0.001**	223.39	672.31	117.93	565.78
Temperature	1.97	58.96	**0.053**	-0.01	0.75	18.58	18.95
Tree cover density	1.70	33.95	0.10	-2.49	27.65	31.31	43.88
NDVI	-1.58	70.26	0.12	-0.09	0.01	0.69	0.65
Distance to the closest path	1.64	28.15	0.11	-4.41	38.20	20.51	43.88

Supplementary Table 6. Linear Mixed Models examining the relationships between alpha diversity and environmental predictors.

Response variable	Predictor	Est	SE	CI		p	Moran's I Test p
Shannon	Distance to city center	0.12	0.09	-0.07	0.31	0.209	0.962
	Temperature	-0.02	0.08	-0.17	0.13	0.812	
	Human presence	0.06	0.07	-0.08	0.2	0.391	
	**Tree cover density**	**0.18**	**0.09**	**0**	**0.36**	**0.045**	
	Distance to the path	-0.02	0.07	-0.15	0.11	0.789	
No of Observed OTUs	Distance to city center	0.63	0.63	-0.63	1.89	0.325	0.33
	Temperature	-0.08	0.5	-1.08	-0.92	0.875	
	Human presence	0.04	0.45	-0.86	0.95	0.925	
	**Tree cover density**	**1.2**	**0.59**	**0.03**	**2.37**	**0.045**	
	Distance to the path	-0.26	0.43	-1.12	0.61	0.553	
Faith's PD	Distance to city center	0.36	0.31	-0.25	0.98	0.245	0.887
	Temperature	-0.01	0.23	-0.47	0.44	0.949	
	Human presence	-0.02	0.21	-0.43	0.39	0.949	
	**Tree cover density**	**0.54**	**0.27**	**0**	**1.08**	**0.049**	
	Distance to the path	-0.09	0.19	-0.47	0.3	0.656	

Supplementary Table 7. Sampling localities and sample size.

Urban/Rural	Habitat Type	Sampling Site	Cavity Type	Initial Dataset		Final Dataset	
				N nests	N samples	N nests	N samples
**Rural**	Natural forest	KPN	Nest box	12	14	12	12
	Peri-urban village	PAL	Nest box	15	16	13	13
**Urban**	Office area	UNI	Nest box	3	3	3	3
	Residential area	MUR	Nest box	5	5	5	5
		OLO	Nest box	1	1	1	1
	Urban forest	BIB	Nest box	11	11	7	7
		BIE	Natural Cavity	8	8	6	6
	Urban park	POL	Nest box	25	27	24	26
	Urban woodland	CMZ	Nest box	8	8	8	8
		LOL	Nest box	1	1	1	1
**Grand Total**				**89**	**94**	**80**	**82**

Supplementary Text 1. Environmental and Spatial Variables Used in the Study

1. Environmental variables

Environmental variables were collected i) on the ground, ii) using Geographic Information System tools (i.e., QGIS) and iii) remote sensing (i.e., digital photography and satellite imagery).

1.1. Environmental variables collected on the ground:

1.1.1. Human presence

Human presence was derived by quantification of all humans (identified as bikers or pedestrians) and dogs (always associated with humans in our study system) within a 15m radius around each nestbox. Specifically, 15-second counts were repeated multiple times at each fixed location (i.e., n = 84 nestboxes) and during three great tit and blue tit breeding seasons (in 2016, 2017 and 2018). Finally, an average index of human presence was derived for any given nestbox. This methodology is further described by Corsini et al. (1, 2). As the data were collected in a different framework, the human presence was not quantified for nests situated in natural cavities (n = 9).

1.1.2. Sound pollution

Sound pollution was recorded on DbC scale using hand-held sound level meters with a microphone (Digital Sound Level Meter SL-200). The sound level was recorded in 4 days throughout the field season and three times per day. A specific track for each study site was assigned to a fieldworker who, stopping 5 seconds at each nestbox location, recorded the highest value of sound pollution. Further details on this methodology are detailed in Szulkin et al. (3).

1.1.3. Temperature

The temperature was obtained from Thermocrones ibuttons DS1921G set with a 1 - hour sampling frequency. The data loggers were active from 24/04/2018 until 30/06/2018. An average temperature was calculated for each termocrone and each nestbox was assigned a temperature value corresponding to the nearest loggers as described in Szulkin et al. (3).

1.2. Environmental variables extrapolated from digital photography and satellite imagery

1.2.1. Light pollution

A 10m pixel resolution map of light pollution in Warsaw was extrapolated from night-time digital photography, shot on 08/10/2015 by astronauts from the International Space Station (4). The light pollution map was uploaded and georeferenced in QGIS (v.3.10.4). Georeferencing was done manually via the "Georeferencer-GDAL" plugin available by default in the software. The averaged value of light pollution was calculated at the nest level for all the sampling locations (n = 93) in a 100m radius buffer in QGIS.

1.2.2. Tree cover density

A 20m pixel resolution map of tree cover density in Warsaw (in a range from 0 to 100%) was downloaded from Copernicus Land Monitoring Services (https://land.copernicus.eu/sitemap). After the tree cover density map was uploaded in QGIS, averaged values measured at the nest level were determined using a 100m radius buffer as described in point 3.1 light pollution.

1.2.3. Normalized Difference Vegetation Index (NDVI)

The Normalized Difference Vegetation Index (NDVI, given in a range from -1.0 to 1.0) is a graphical indicator of live green vegetation. It was estimated using satellite images derived from SENTINEL2 and available on the Earth Explorer website (https://earthexplorer.usgs.gov). Ten-meters pixel resolution satellite images were downloaded for two different bands: 4 and 8 (corresponding to “Red” and “Near-Infrared” as RED and NIR, respectively). Both images were then uploaded in QGIS, where NDVI was computed through the following formula via Raster calculator tool :

NIR - RED / NIR + RED in bands 8 - 4 / 8 + 4

After computing the NDVI for the full map of Warsaw, averaged NDVI values at the nest level were estimated in a 100 m radius buffer as described in 3.1.

2. Spatial Variables

2.1. Spatial variables collected using GIS

Nestbox distances to the closest road and closest path were measured in meters using the “Measure line” tool in QGIS as described in Corsini et al. (1). Roads were identified as paved infrastructural networks used by motorized vehicles. Paths included paved and unpaved walkways used by pedestrians.

Nestbox distances to the city centre (i.e. Palace of Culture and Sciences location - 52°13′54″N, 21°00′23″E) were measured in meters using the “Measure line” tool in QGIS.

Furthermore, the distance matrix of the geographical coordinates of the sampling sites was calculated based on Haversine distance.

References

1. M. Corsini, A. Dubiec, P. Marrot, M. Szulkin, Humans and tits in the city: Quantifying the effects of human presence on great tit and blue tit reproductive trait variation. *Front. Ecol. Evol.* (2017) https:/doi.org/10.3389/fevo.2017.00082.
2. M. Corsini, P. Marrot, M. Szulkin, Quantifying human presence in a heterogeneous urban landscape. *Behav. Ecol.* (2019) https:/doi.org/10.1093/beheco/arz128.
3. M. Szulkin, *et al.*, “How to Quantify Urbanization When Testing for Urban Evolution?” in *Urban Evolutionary Biology*, (2020) https:/doi.org/10.1093/oso/9780198836841.003.0002.

4. C. C. M. Kyba, *et al.*, High-resolution imagery of earth at night: New sources, opportunities and challenges. *Remote Sens.* (2015) https:/doi.org/10.3390/rs70100001.
